# Habituation of the electrodermal response – A biological correlate of resilience?

**DOI:** 10.1371/journal.pone.0210078

**Published:** 2019-01-25

**Authors:** Frederick R. Walker, Ashley Thomson, Kane Pfingst, Elke Vlemincx, Eugene Aidman, Eugene Nalivaiko

**Affiliations:** 1 University of Newcastle, Newcastle, Australia; 2 Australian Army, Canberra, Australia; 3 Queen Mary University of London, London, United Kingdom; 4 DST Group, Adelaide, Australia; Icahn School of Medicine at Mount Sinai, UNITED STATES

## Abstract

Current approaches to quantifying resilience make extensive use of self-reported data. Problematically, this type of scales is plagued by response distortions–both deliberate and unintentional, particularly in occupational populations. The aim of the current study was to develop an objective index of resilience. The study was conducted in 30 young healthy adults. Following completion of the Connor-Davidson Resilience Scale (CD-RISC) and Depression/Anxiety/Stress Scale (DASS), they were subjected to a series of 15 acoustic startle stimuli (95 dB, 50 ms) presented at random intervals, with respiration, skin conductance and ECG recorded. As expected, resilience (CD-RISC) significantly and negatively correlated with all three DASS subscales–Depression (r = -0.66, p<0.0001), Anxiety (r = -0.50, p<0.005) and Stress (r = -0.48, p<0.005). Acoustic stimuli consistently provoked transient skin conductance (SC) responses, with SC slopes indexing response habituation. This slope significantly and positively correlated with DASS-Depression (r = 0.59, p<0.005), DASS-Anxiety (r = 0.35, p<0.05) and DASS-Total (r = 0.50, p<0.005) scores, and negatively with resilience score (r = -0.47; p = 0.006), indicating that high-resilience individuals are characterized by steeper habituation slopes compared to low-resilience individuals. Our key finding of the connection between habituation of the skin conductance responses to repeated acoustic startle stimulus and resilience-related psychometric constructs suggests that response habituation paradigm has the potential to characterize important attributes of cognitive fitness and well-being–such as depression, anxiety and resilience. With steep negative slopes reflecting faster habituation, lower depression/anxiety and higher resilience, and slower or no habituation characterizing less resilient individuals, this protocol may offer a distortion-free method for objective assessment and monitoring of psychological resilience.

## Introduction

Psychological resilience has been defined as the process of positive adjustment to adverse and potentially traumatic events [1]. Resilient individuals are known to exhibit positive adaptation when they encounter significant adversity, tragedy, threat, or other sources of stress [2]. The term “resilience” was first used in this context by Block [3]; however the concept could be traced back to Freud’s theory of personality where it had a name of “ego strength” [4]. In the context of exposure to adverse or traumatic events, an indicator of resilience would be the absence of psychiatric disorder symptoms, such as post-traumatic stress disorder (PTSD). This explains why there has been a growing interest in resilience in at risk populations, most notably first responders and military personnel (eg. [5], [6]). There is extensive literature covering psychobiology of resilience (see [7] for review); it provides substantial evidence that anxiety and depression are closely linked with psychological resilience, and with disorders that arise from a lack of psychological resilience, such as PTSD [8].

Despite the obvious potential, implementation of resilience training and monitoring programs has proven difficult, in large part because of the absence of accurate and rapid tools to objectively assess resilience. Presently, psychometric approaches dominate the research landscape. Apart from inherent limitations due to its subjective nature, they can suffer from a serious problem known as self-report bias [9]. Self-report bias refers to the situations in which participants want to respond in a way that makes them ‘look as good as possible’. As a result, participants can under-report items that they believe would be considered inappropriate by evaluators, and over-report items viewed as appropriate. It has been convincingly demonstrated that self-report bias is extremely likely when employees believe there is at least a remote possibility that their employers could gain access to their responses [9]. Not surprisingly, self-report bias is also strongly expressed in military settings [10].

An ideal solution to the inherent limitations of self-report measures of resilience would development of validated physiological and/or biological predictors of resilience. Such readouts would be significantly less prone to manipulation and therefore offer the possibility of improved assessment accuracy. In an attempt to address this problem, we recently performed a comprehensive systematic review of biomarkers of resilience [11]. We concluded that among the number of physiological, biochemical and immunological markers, the most promising resilience-related index is the speed (slope) of habituation of acoustic startle response, and especially of its sudomotor component–electrodermal response. The latter reflects transient stress-induced increase in sweat release on palms of the hand and soles of the feet, and has been extensively studied by psychophysiologists [12]. The response could manifest in two ways–as a change in voltage between two cutaneous electrodes (galvanic skin response, GSR) or as an increase in the skin conductance level (SCL) between them; in the following text, we use “electrodermal response” in the latter meaning.

Patients with post-traumatic stress disorder (PTSD) represent, by definition, a low-resilient group in the general population. Startle responses have been extensively studied in this group, and the currently established view is that abnormal features of these responses comprise elevated eyeblink EMG [13–16], larger tachycardia [16–18] and higher and slower habituating skin conductance responses (SCR) [16, 18]. Many studies, however, have reported lack of potentiated eyeblink in PTSD patients [17–19], and after performing a comprehensive analysis of existing publications, Pole [20] concluded that under more stringent tests for significance, PTSD was reliably related only to higher HR response and to slower SCR habituation. Moreover, the established view has been further challenged by [21] who concluded that elevated startle responses reflect not hyperarousal *per se*, but rather the process of fear potentiation. Indeed, elevated startle responses in PTSD patients compared to controls were found in those studies where the level of fear was manipulated by laboratory stressors [13, 22]. From these studies it is difficult to conclude whether slow habituation to repetitive arousing stimuli is a part of PTSD symptomatology, or whether it is a pre-existing phenotype element that could be potentially associated with vulnerability to psychological stressors. We argue that if the latter is true, then in a mentally healthy population, with little motivation to mask their internal states, the psychometrically-assessed resilience scores will have a negative association with habituation of electrodermal response, i.e. that high-resilient individuals will habituate faster. Accordingly, we have designed our experiments to test this hypothesis.

Our secondary aim was to explore the relationship between cardiac and respiratory parameters and resilience in healthy subjects. Existing evidence relating cardiovascular reactivity to resilience is controversial (see Discussion), while heart rate variability-based results are more consistent (see [11] for review). There is no data on relationship between respiratory measures and resilience, and we focussed on three indices–respiratory intervals at baseline, stress-induced tachypnoea and sigh frequency. Sighs have been considered functional in the regulation of stress and emotions [23]. Sighs frequency during stress exposure is expected to increase with increasing stress regulation [23]. Whereas increased stress regulation during stress exposure would be a marker of high regulatory capacities and high resilience, increased stress regulation during non-stressful resting periods, possibly due to hypervigilance and/or hyper-reactivity to potential contextual stressors, would be indicative of maladaptive regulation and low resilience. Consistent with the former, increased sigh frequencies have been found during mental arithmetic stress [24, 25] and negative and/or high arousal emotions in healthy persons [26]. Consistent with the latter, excessive sigh rates during baselines have been reported in PTSD [27] and other anxiety disorders [28–33]. We thus attempted to reveal whether there is any association between individual resilience level and sigh frequencies during mental arithmetic in a healthy sample.

## Materials and methods

### Participants and experimental procedures

The study was conducted in 30 young healthy volunteers of both genders (17 males and 13 females) aged 18–40 (26±4) y.o. The study protocol was approved by the University of Newcastle Human Research Ethics Committee. Inclusion criteria were good overall physical and mental health. On arrival to the lab, participants signed the informed consent and filled two psychometric scales: CD-RISC for rating resilience level [34] and DASS for rating state depression, anxiety and perceived life stress [35]. Subjects were subsequently fitted with gel-filled Ag/AgCl electrodes for recording ECG and skin conductance, and with a respiratory belt with piezo-electric sensor (ADInstruments, Australia) for recording respiratory movements. Skin conductance electrodes were placed on the palmar surface of the index and middle fingers of non-dominant hand and connected to the skin conductance recording unit (Model 2701, UFI, USA).

Baseline recordings of physiological variables were conducted for 5 min while participants were instructed to watch a neutral image on a computer screen. They were then asked to perform modified mental arithmetic task (consecutively subtracting of 13 from a 4-digit number); in order to prevent effects of speech on respiration, they were instructed to type the results in a computer data pad instead of counting aloud. During this task, 15 acoustic stimuli (computer-generated white noise, 50 ms, 95 dB) were presented via stereo headphones at random intervals ranging 30–50 s. Recordings of ECG, respiration, finger skin conductance and acoustic stimuli were performed using PowerLab 8s A/D convertor and Chart v.7 software (ADInstruments, Sydney, Australia). Data were sampled at 1 KHz for ECG and 100 Hz for the respiratory and SCL signals.

### Data analysis

ECG R-R interval were used for computing heart rate (HR); for determination of vagal heart rate variability (HRV) indices, we employed the HRV module of LabChart Pro v.8.0. These measures included the standard deviation of the R-R interval (SDNN, ms), the root mean square of successive R-R interval differences (RMSSD, ms) and the high frequency (HF) HRV power (frequency band 0.15–0.4 Hz; ms^2^). Respiratory rate was computed from the intervals between peaks of the respiratory signal. For each subject, sigh frequency during mental arithmetic was calculated as the number of breaths with a relative inspiratory volume at least twice the mean inspiratory volume during mental arithmetic (Ramirez, 2014; Vlemincx et al., 2013; Wilhelm et al., 2001).). The latencies of the SCL responses to acoustic stimuli were determined in seconds as time between the onset of sound and an onset of the SCL response; their amplitudes (in μS) were quantified as a difference between 2 s pre-stimulus baseline and the maximum of the following response. Only SCL changes that occurred within 1–4 s post-stimulus were included in the analysis.

Statistical analysis was performed using Prizm v.6.0 (GraphPad, USA). Associations between autonomic variables and psychometric scores, and between different psychometric scores were assessed using Pearson correlation. Given the relatively small sample size and the pilot nature of this study, we chose not to adjust for multiple statistical tests, in order to avoid increasing type II error. Effects of mental arithmetic task on autonomic variables were determined by means of paired t-tests. A regression analysis was performed to investigate the effects of each psychometric score on sigh frequency, while controlling for the other scores. For assessing habituation of the SCL responses, they were initially normalized for each individual (i.e. first response = 100%), plotted against the trial number, and then habituation slope was determined by linear regression. P<0.05 was accepted as a criterion for statistical significance. Data values are reported as Mean ± SEM.

## Results

### Psychometric scores and their inter-relationship

**Fig 1:**
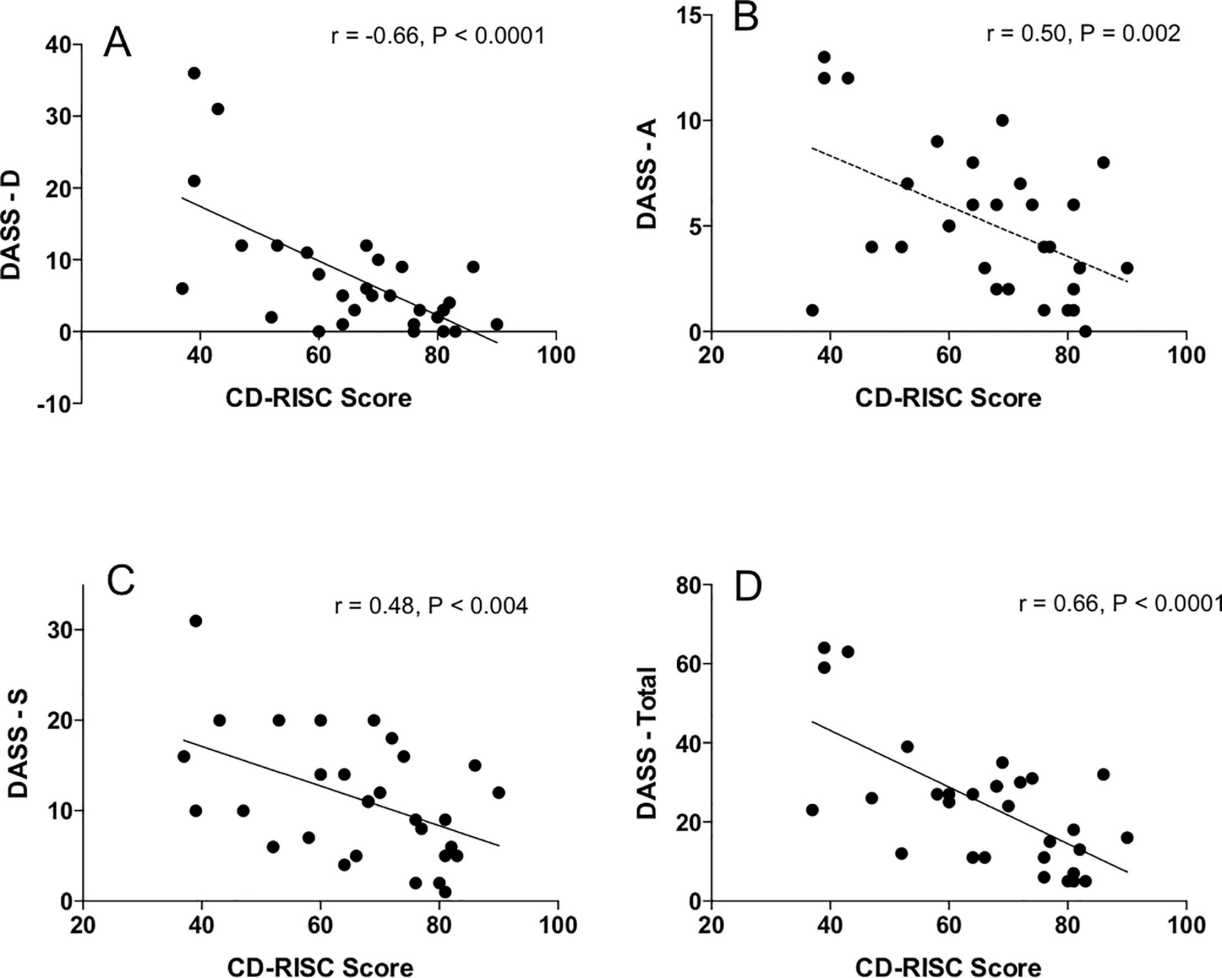
Relationships between DASS subscales scores and resilience scores. There was significant inverse correlation between resilience and Depression (A), Anxiety (B), Stress (C) and Total (D) DASS self-rating measures.

We found the following mean values for the self-rated psychometric scales: resilience (CD-RISC): 65.1±2.8; DASS-Depression: 7.4±1.6; DASS-Anxiety: 5.2±0.6; DASS-Stress: 11.3±1.2 and DASS-Total: 24.2±2.9. Significant negative correlation was observed between CD-RISC Resilience scores and Depression (r = 0.66, p<0.0001), Anxiety (r = 0.50, p<0.005) and Stress (r = .48, p<0.005) subscales of the DASS test, as well as with the DASS Total score (r = 0.66, p<0.0005) (Fig 1).

### Autonomic changes provoked by mental arithmetic task and their relations to psychometric scores

**Fig 2:**
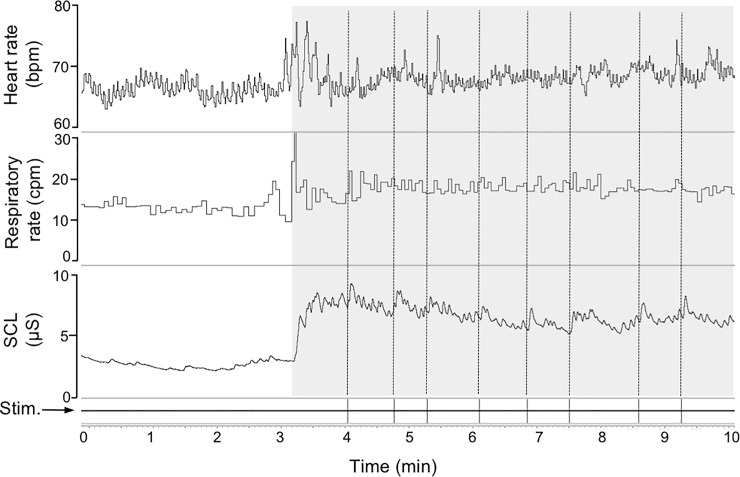
Representative recording of heart rate, respiratory rate and skin conductance level in one subject before and during presentation of acoustic stimuli (Stim.) on the background of mental arithmetic task; shaded area corresponds to the task. Note consistent SCL responses to startle stimuli, without accompanying cardiac or respiratory responses; and reduction of the respiratory sinus arrhythmia during the task.

An example of data recording from one subject is presented in Fig 2. Mental arithmetic test (MAT) caused significant reduction of the ECG R-R interval (from 780±32 at baseline to 756±26 ms, p<0.05) resulting in increased HR (from 79±3 to 82±3 bpm, p<0.05). This was associated was observed with a decline in the SDRR, a vagal HRV index (from 62±6 to 54±5 ms, p<0.05) and with a trend towards the reduction of the high-frequency (HF) HRV power (from 1457±437 to 1181±320 ms^2^, p = 0.058). In addition, HF HRV power negatively correlated with CR-RISK resilience (r = -0.30, p = 0.06) and DASS-depression (r = -30, p = 0.058), though neithrer of the two correlations reached statistical significance.

**Fig 3:**
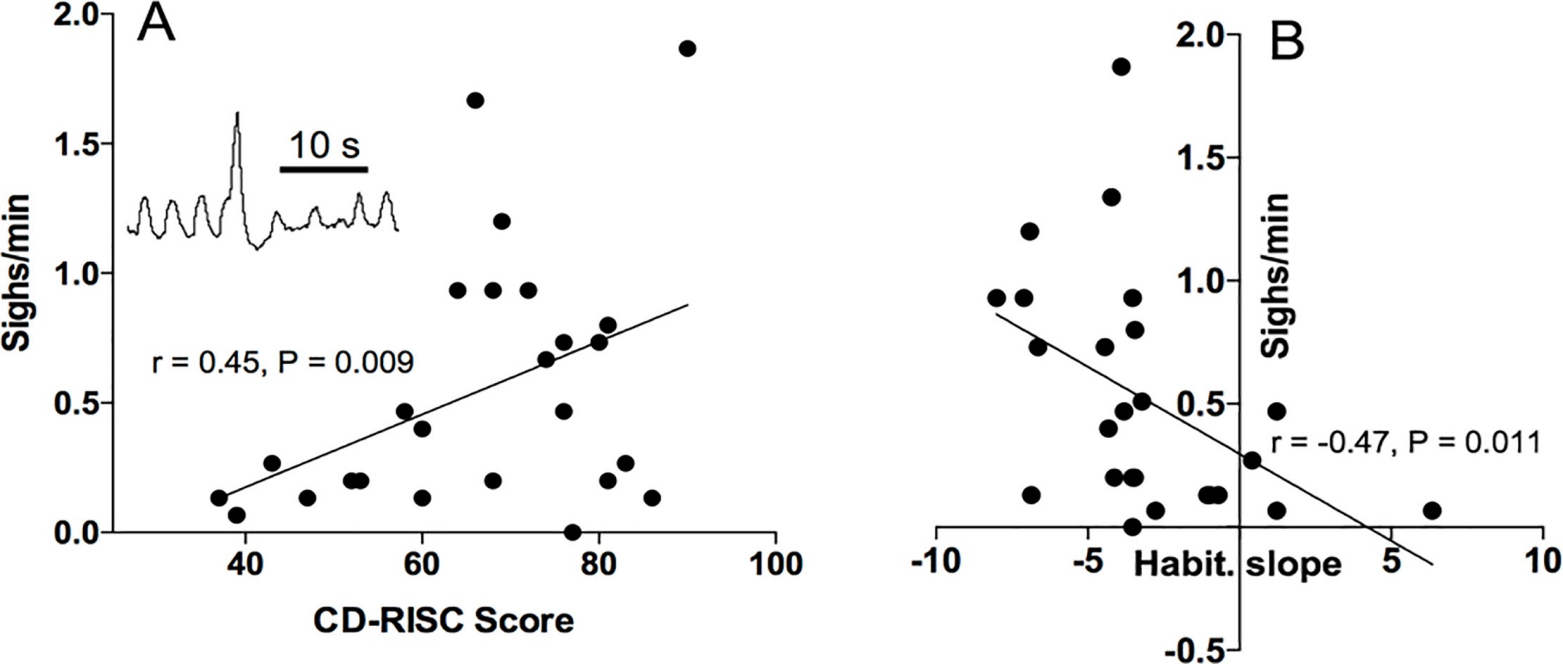
Relationships between sigh frequency during mental arithmetic task and resilience score (A) and habituation slope of startle responses F(B). Inset in (A) shows typical sigh morphology.

Significant reduction in the respiratory interval was observed during the Mental Arithmetic Task (from 3.3±0.2 at baseline to 2.9±0.71 during stress, p<0.05) resulting in increased respiratory rate (from 17.1±1 to 21±1 cpm, p<0.05; Fig 2). Baseline respiratory cadence tended to correlate with CD-RISK resilience (r = 0.3, p = 0.061), but no association between MAT-induced tachypnoea and resilience score was observed. We also focussed on the relationship between sigh frequency and psychometric scores. Due to movement artefacts, it was not possible to accurately identify and count sighs during control period, and we thus limited our analysis to the test period. Mean sighing frequency was 0.48±0.09 sighs/min (range 0–1.8 sighs/min). A significant positive correlation between Sigh frequency and CD-RISC Resilience score was observed (r = 0.45, p = 0.01; Fig 3A) and significant negative correlation between sigh frequency and habituation slope of electrodermal responses (r = -0.47, p = 0.0501; Fig 3B; see next section for the description of startle habituation). Regression analyses revealed that CD-RISC scores significantly improved the prediction of sigh frequency during MAT, over and above the DASS scores (β = 0.02, p = 0.011), whereas DASS scores had no effects on sigh frequency beyond CD-RISC (β = 0.00004, p = 0.99; b = -0.01). In other words, the number of sighs per minute during MAT increases by .02 as CD-RISC scores increase one unit, while controlling for collinearity between CD-RISC and DASS.

**Fig 4:**
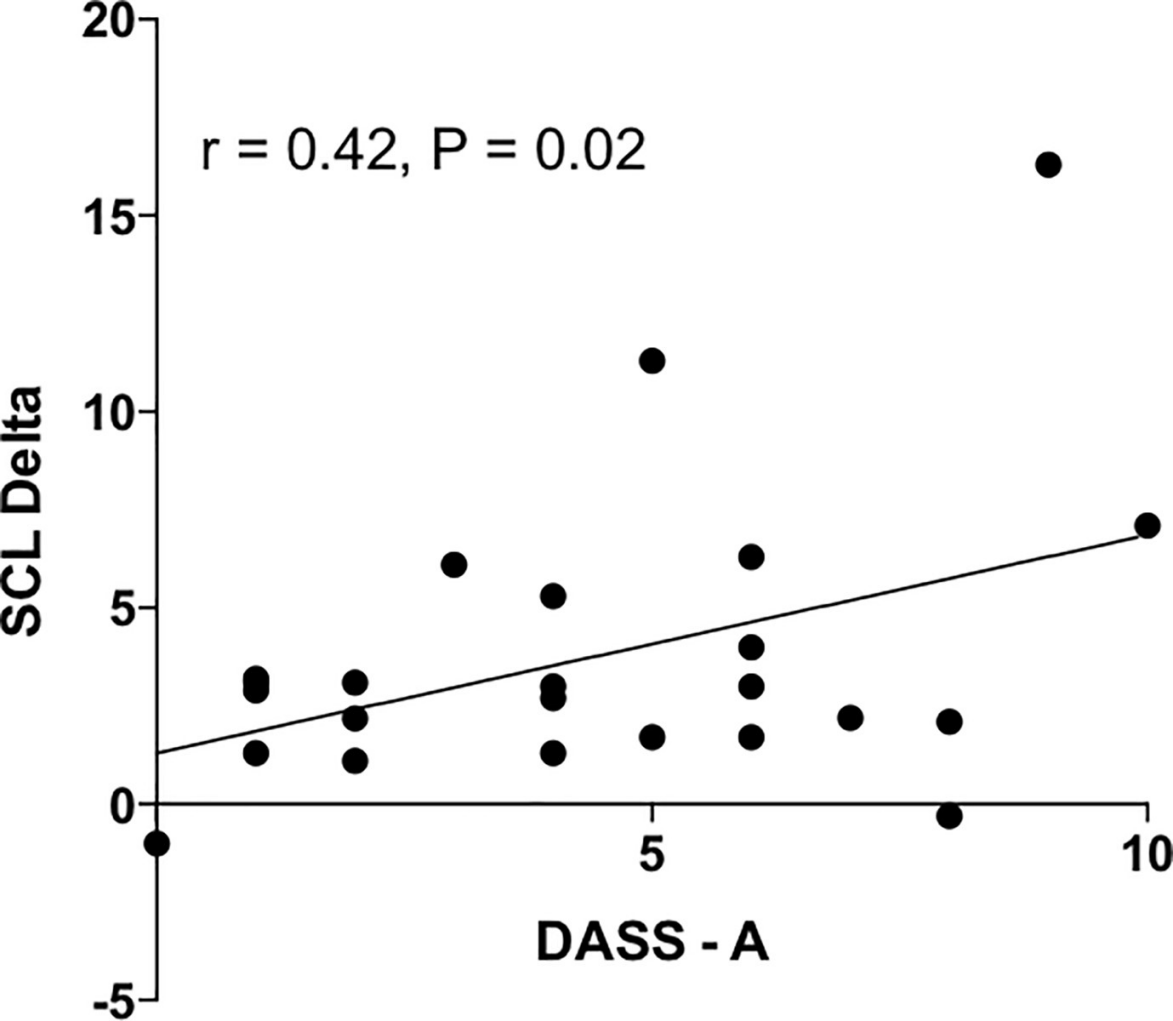
Relationship between increase in skin conductance level during mental arithmetic task and the Anxiety score of the DASS scale.

Mental arithmetic test also caused consistent increase in skin conductance level (from 10.7 to 14.2 μS (p<0.005; Fig 2). There were no association between baseline SCL and any of psychometric scores. There was however significant positive correlation between the increase in SCL during the test and anxiety score (r = 0.42,p<0.05;Fig 4).

### Autonomic responses to acoustic startle stimuli

**Fig 5:**
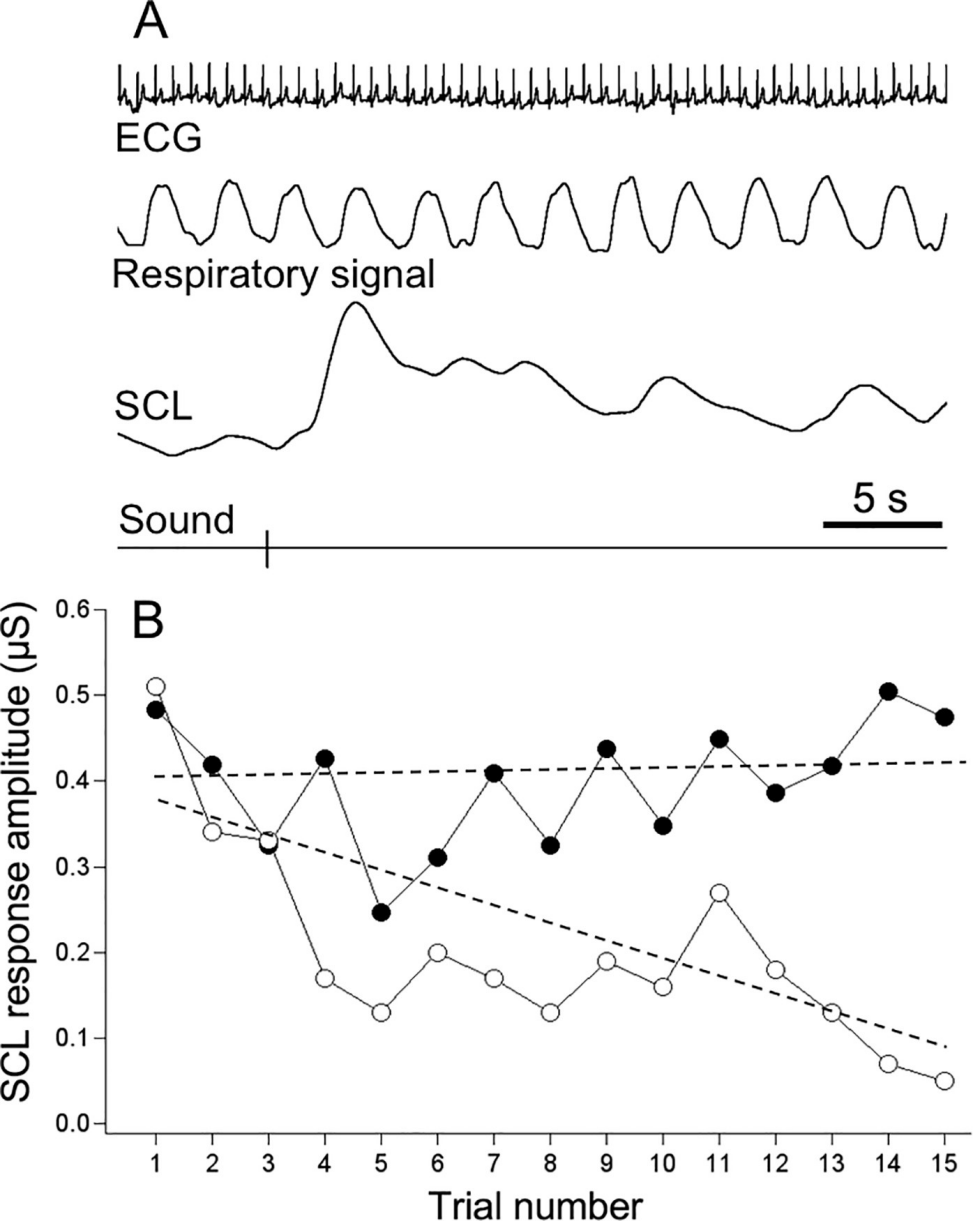
Habituation of skin conductance (sweating) responses to acoustic startle stimuli can discriminate between high- and low-resilient individuals. A–raw data records of ECG, respiration and skin conductance level (SCL) during presentation of acoustic startle stimulus (sound; 50 ms, 95 dB). B–pattern of habituation of SCL responses in one high-resilient (CD-RISC score = 78; open circles) and one low-resilient (CD-RISC score = 39; closed circles) individual. Dashed lines represent habituation slopes determined by linear regression.

Acoustic startle stimuli consistently provoked transient SCL responses in all participants; Fig 5A illustrates an example of such response. Data from two participants were excluded due to equimpent malfunction. For the first SCL response, mean latency was 2.34±0.16 s, and mean amplitude was 1.56±0.35 μS. Neither of these two measures correlated with any of psychometric scores. While we noted occasional small heart rate responses to acoustic stimuli, they were not consistent within individuals as confirmed by signal averaging; we also did not find any indications for respiratory responses.

**Fig 6:**
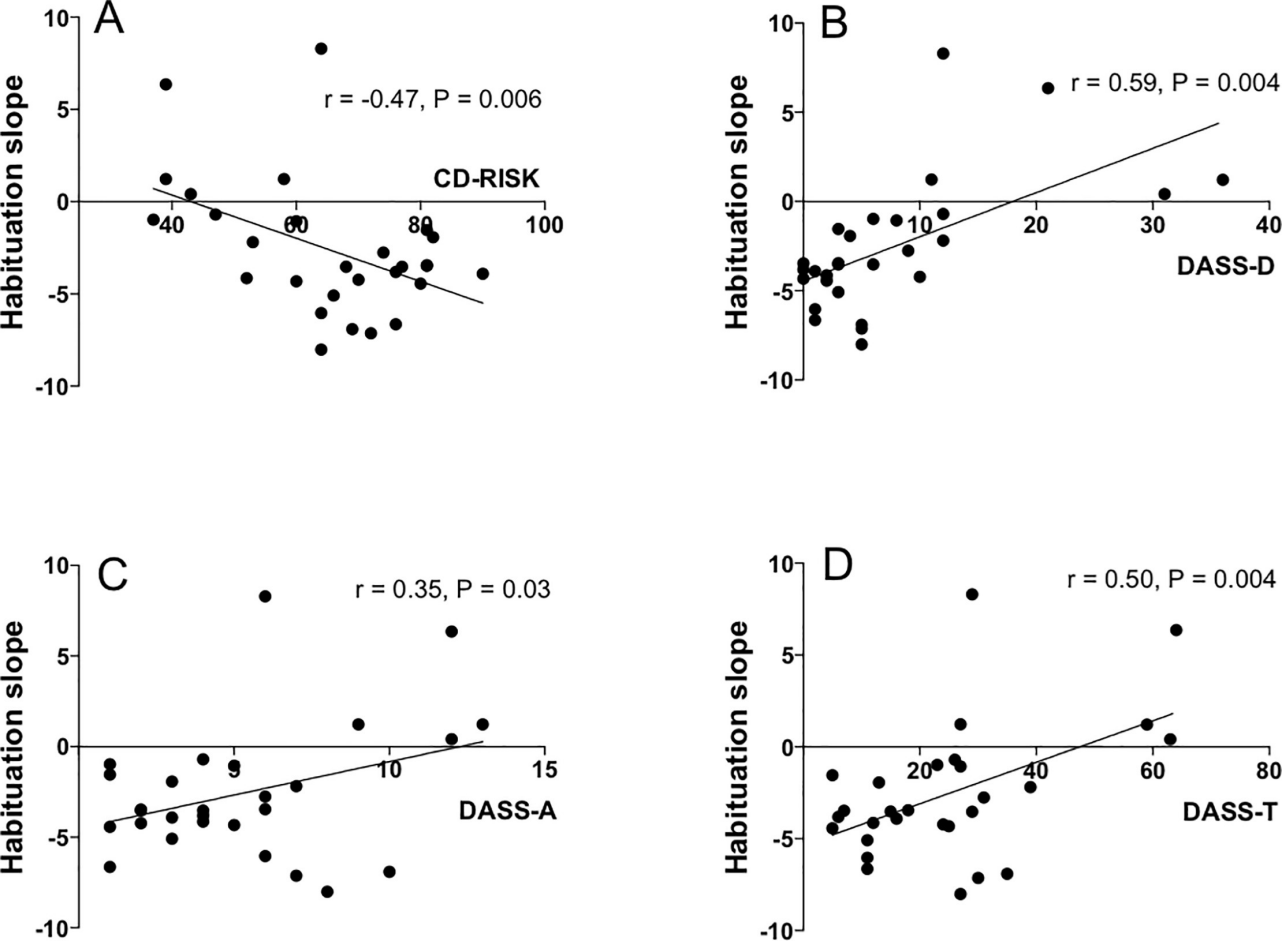
Relationships between habituation of the SCL component of startle response and psychometric scores. There was significant inverse correlation between the habituation slope and resilience score (A) and significant positive correlation between the habituation slope and Depression (B), Anxiety (C) and Total (D) scores of the DASS scale.

In order to characterise habituation of SCL responses (reduction in amplitude with repeted stimulation) and to perform inter-individual comparison, we regressed SCL amplitude on trial number for each participant, and determined the slope of the linear fit (Fig 5B). While conducting this analysis, we noted that in high-resilient individuals, habituation occurred faster compared to low CD-RISC scorers. This finding was confirmed statistically by a significant negative correlation between the habituation slope and CD-RISC resilience score (r = -0.47; p = 0.006; Fig 6A). We also found significant positive correlation between the habituation slope and DASS scores (r = 0.50, p < 0.005 for the total score, r = 0.59, p < 0.005 for Depression r = 0.35, p < 0.05 for Anxiety; see Fig 6C and 6D). In contrast, latency of SCL responses was not affected by the trial number (data not shown).

Subsequent analysis of partial correlations suggests that the observed associations between habituation slope of the SCL component of startle response and DASS scores is driven by its Depression subscale (DASS-D): DASS-D is a significant predictor of the habituation slope (B = 0.308, t = 3.13, p = 0.004) while DASS-A does not add to the prediction beyond DASS-D (B = -0.206, t = -0.827, p = 0.416 for the second step in a hierarchical regression following DASS-D in the first step). Similarly, while CD-RISK on its own is predictive of the habituation slope (B = -1 .738, t = 2.472, p = 0.02, collinearity tolerance = .674), it does not add to the prediction beyond DASS-D: (B = -0.015, t = -0.28, p = 0.78 for the second step in a hierarchical regression following DASS-D in the first step).

## Discussion

A major novel finding of our pilot study is the demonstration of a robust association between habituation of the SCL responses to acoustic startle stimuli and several psychometric measures, namely negative correlation of habituation slope with resilience and positive association with trait depression and anxiety. As more negative slopes reflect faster habituation, this means that slower or no habituation was observed in less resilient individuals and in those with higher depression and anxiety scores. Correlational analysis of psychometric scores also revealed that low-resilient individuals scored high on depression, anxiety and life stress scales. Our supplementary analyses showed that this pattern of correlations is driven by depression as a key predictor of our habituation slopes. The pilot nature of this study has allowed us to keep the constructs of Anxiety, Stress and aggregated DASS in the overall pattern for completeness. However, this may have inflated the risk of type I error, and as a result, our findings should be considered preliminary and subject to validation in subsequent fully-powered investigations.

### Relationship between psychometric measures

Despite their subjective nature, psychometric instruments such as questionnaires and scales have long been established as preferred method for assessing individuals’ psychological status. Although the main focus of this study was to assess psychological resilience with the aim of predicting it using physiological methods, we have used a combination of questionnaires in full knowledge that using the Connor-Davidson Resilience Scale alone would not provide a clear view of the bigger picture. There is substantial evidence that anxiety and depression are closely linked with psychological resilience in both directions, and with disorders that arise from a lack of psychological resilience, such as PTSD [8]. This necessitated the use of another psychometric instrument that gives a score for depression and anxiety with consistent and reliable results across all subjects, therefore the clinical favourite Depression and Anxiety Stress Scale (DASS) was used [36, 37]. The main purpose of this was to determine if any relationship existed specifically with the resilience scores used in this study, and also the nature of the relationship. It also served to ensure that none of the subjects had significantly high depression and/or anxiety, firstly to minimise any potential psychological harm that may arise from participation, and secondly to reduce the likelihood of skewing the data. No subject scored too high in any of the DASS sub-scales so no one was excluded from participation or data analysis.

Intercorrelations between DASS subscales and CD-RISC resilience scores were all significant (see Table 1). Interestingly, depression appeared to have a stronger relationship with resilience than anxiety, which was somewhat unexpected since anxiety symptoms are more frequently associated with a lack of coping mechanisms [7], and the neurobiology associated with anxiety disorders has more frequently been associated with low resilience compared to major depression [38]. This relationship has been highlighted in studies such as [39], which assessed life satisfaction, depression, anxiety and resilience across the life span of men. They concluded that the emergence of a crisis in midlife manifested by health concerns was accompanied by anxiety and reduced resilience. Our results are also consistent with similar findings [40] of the relationship between depression, anxiety, stress and resilience. Together, these observations are consistent with the patterns observed in our psychometric data.

**Table 1:** Intercorrelations between the study variables.

	CD-RISC	DASS-D	DASS-A	DASS-S	DASS-Total	Sex	Age
**DASS-D**	-0.66***						
**DASS-A**	-0.50**	0.74***					
**DASS-S**	-0.48**	0.45*	0.59***				
**DASS-Total**	-0.67***	0.88***	0.85***	0.79***			
**Sex**	0.06	-0.13	-0.16	0.18	0		
**Age**	-0.03	-0.26	-0.25	-0.03	-0.23	-0.12	
**Habituation slope**	-0.47*	0.59***	0.35	0.25	0.50**	0.09	0.02

*p < 0.05

**p < 0.01

p*** < 0.005

### EDR habituation and resilience

Electrodermal component of the orienting/startle response is one of the most studied psychophysiological response types, and its habituation with presentation of repetitive stimuli has been described in many studies conducted in healthy individuals (e.g. [41]). For the purpose of our discussion, however, the major interest represents results obtained in patients with PTSD who by definition fall into the low-resilient category. As we already noted in the Introduction, thorough analysis of existing publications revealed that slow habituation of the EDR component of a startle response is a commonly reported abnormality in PTSD patients (see [20] for review). The most impressive case here is the study by [42] where EDR recorded in subjects with PTSD did not habituate at all. Our results are consistent with the existing evidence and extend the previous findings by demonstrating that EDR habituation is associated with the severity of sub-clinical symptomology (as measured by CD-RISC). The specificity of EDR habituation as a marker of post-traumatic symptomology requires further examination, given that our results indicate similar associations with Anxiety and Depression.

A major question in PTSD research is whether differences in startle-induced responsivity between resilient and vulnerable individuals are innate and exist pre-trauma or they represent the result of trauma-induced neural remodeling. As we observed significant negative association between resilience scores and the EDR habituation rate in our healthy subjects, we are inclined to favor the idea that startle differences can exist pre-trauma. Generally, habituation is defined as the reduction of a response during repeated presentations of a stimulus. It is regarded as a form of non-associative learning and represents one of the mechanisms underlying formation of implicit memory [43]. Neural mechanisms responsible for habituation were described in detail in invertebrates [44]; in mammals these mechanisms are more complex and include central nervous gating that operates to dampen unnecessary responding to innocuous stimuli [45].

It will be of interest for future studies to examine whether slow startle habituation potentially reflects, at a very basic neural level, lower cognitive flexibility–an established vulnerability factor for mental health [46]. Indeed, habituation could be regarded as a form of learning, and the ability to rapidly classify new stimulus as non-aversive/non-salient, without triggering unnecessary physiological responses, appears to be a more advanced stress-coping strategy. The idea that slower habituating of EDR to startle stimuli may be an innate vulnerability factor for PTSD is further supported by lack of differences in habituation slopes found in monozygotic twins discordant to combat exposure/PTSD [47]. An additional and even stronger evidence is represented by one of the very few prospective studies where EDR habituation was found to be a predicting factor for subsequent development of PTSD symptoms in police officers [22].

### Methodological considerations

In studies employing acoustic startle paradigm, parameters of acoustic stimuli vary, in both sound level (from 65 to 105 dB) ant type (white noise vs. pure tone). As these parameters might to some extent affect the outcome, in our experiments we replicated the protocol used by Shalev’s study [42] where clear lack of EDR habituation was found in patients with PTSD. Next, as presenting acoustic stimuli on the background of the mental arithmetic stress could be considered as limitation of our study, we would like to comment on why we have assumed this approach. This was done for two purposes: firstly, to standardize the mindset of participants so that they were concentrated on the external task, and this must reduce variability from not attending/attending the sound stimulus [41]. Secondly, [13, 22] have demonstrated that stressful context (threat of electric shocks) was essential for non-habituating pattern of the EDRs. For ethical reasons, we did not include in our study electric shock, and have chosen mental arithmetic instead. In full accord with numerous previous studies (eg. [48, 49], increased skin conductance level, heart rate and reduced vagal HRV indices confirmed that this intervention was indeed to some extent stressful. This was further supported by substantially elevated respiratory rate during the task. Interestingly, relative increase in respiratory rate (+24%) was much more prominent compared to relative tachycardia (+4%) during mental arithmetic task, replicating previous studies [48, 50] and confirming the view that respiratory indices are closely associated with affective states [51]. Finally, most previous studies of startle response reported small rises in heart rate, usually in the range of 1–2 bpm. We propose that lack of consistent cardiac responses in our experiments was probably due to the masking effect of small steady tachycardia produced by mental arithmetic.

### Cardiorespiratory measures and resilience

We did not find any association between HR at baseline or stress-induced tachycardia and resilience score. This is not surprising providing controversy in the literature related to this question: while some studies reported no difference between PTSD patients (who by definition fall into a low-resilient category) and healthy controls in cardiac responses mental arithmetic or cold pressor test [52–55], others found that PTSD was associated with reduced cardiac reactivity [56–58]. Interestingly, blunted cardiac reactivity to psychological stress prospectively predicted symptoms of depression in a large population study [59]. Most studies that addressed the link between HRV and resilience documented association of low cardiac vagal tone with PTSD symptoms (see [60, 61] for reviews). Lack of such association in our participants suggests that vagal tone remains intact even in susceptible (but mentally healthy) individuals.

We are not aware of prior attempts to explore relationships between respiratory pattern and resilience. Our preliminary analysis of respiratory data did not reveal any association between the two. Respiratory regulation is a very complex multifactorial process, and if such association exists, it may be that more sophisticated analytic tools are required for its demonstration.

The present results also show that increased sigh frequencies during a mental arithmetic stressor are positively related to self-reported resilience and to its putative biomarker described in our article. Persons who report to have better stress coping and show stronger electrodermal habituation in response to stress, sigh more during stress. This finding suggests that sighs are functional in stress and emotion regulation, and indicative of adaptive regulation. There are various ways in which sighs may be functional to regulate stress and emotions. Sighs temporarily induce subjective and physiological relief [62, 63], and reinstate healthy, adaptive breathing regulation, by restoring breathing variability [24, 25, 62], lung compliance and blood gas levels [64–67]. Although increased sighing during stress exposure may be adaptive, increased sighing in response to perceived stress in the absence of stress due to hypervigilance and/or hyper-reactivity to potential stress cues in a safe environment (as seen in PTSD) may be maladaptive. Future research could further explore the relations between resilience and excessive sighing in the absence of stress.

## Conclusions

Our study provides evidence that measuring habituation to acoustic startle stimuli might represent a fast, cost-effective and simple test for assessing personality traits such as resilience, depression and anxiety. The ability to identify low-resilient individuals at the stage when their mental health is not impaired would represent a major organizational and financial benefits for health system as they could be selectively subjected to preventative resilience-enhancing psychoeducational programs. It is questionable whether our test could discriminate between these three items, and thus additional work is needed to clarify this and to determine whether integration of several biomarkers would be an advantage. Furthermore, limitations of the current pilot study include the small sample size and it will be thus of major importance for future work to validate the test on larger population. Another important task would be to test whether physiological measures are superior to psychometric scales in assessing resilience; one way to achieve this would be to compare both approaches for the ability to predict PTSD in the longitudinal studies of the at-risk population groups, such as deployed military personnel or police forces.

## Supporting information

S1 Table Individual psychometric, demographic and physiological data from the current study. “Qestionnaires” sheet contains psychometric and demographic data; “SC latency” and “SC amplitude” sheets–data values for latencies and amplitudes, respectively, of 15 electrodermal responses; “HRV baseline” and “HRV test” sheets–data values of HRV parameters at baseline and during mental arithmetic task, respectively; “Respiratory” sheet–data values for respiratory rate and sigh frequency.
(XLSX)

S2 Table Individual electrodermal responses to acoustic stimuli. “GSR Abs” sheet provides absolute values (in μS); “GSR Norm” sheet–normalized values, with magnitude of the 1^st^ response representing 100%.
(XLSX)
